# Yoga for myofascial pain of masticatory muscles – a development and feasibility study

**DOI:** 10.1038/s41405-025-00377-x

**Published:** 2025-12-10

**Authors:** Priya Thimma Ravindranath, Iven Klineberg, Manish Bhutada

**Affiliations:** 1 Faculty of Dentistry, The University of Sydney, Sydney, NSW Australia; 2 School of Dentistry, Faculty of Medicine and Health, The University of Sydney, Sydney, NSW Australia; 3 Present Address: Wolfson Sensory, Pain and Regeneration Centre, IOPPN, King’s College London, London, UK

**Keywords:** Maxillofacial surgery, Oral diagnosis

## Abstract

**Objective:**

Myofascial pain of masticatory muscles or jaw muscle pain, a common component of temporomandibular disorders (TMD) often comes with substantial burden and high healthcare expenses. The aim of this study is to evaluate the feasibility and acceptability of yoga in students experiencing myofascial pain.

**Methods:**

Prospective participants were identified through jaw health screening. 25 students diagnosed with myofascial pain (Research Diagnostic Criteria for TMDs) volunteered for this randomized controlled study. Participants were randomized to one of two interventions for 28 days: either a yoga-inclusive management programme or an active control standard care. The clinical assessors were unaware of the assigned intervention. The primary outcomes were feasibility (adherence) and acceptability (perceived helpfulness and willingness to continue). Secondary exploratory outcomes included pain intensity and distribution, jaw function, oral health–related quality of life, psychosocial measures, and clinician-assessed palpation tenderness.

**Results:**

18 (8 yoga; 10 controls) participants completed the study. Adherence was significantly higher in the yoga group (85%) compared with controls (68%, *p* = 0.048). Yoga participants rated greater perceived benefit (mean 6.3 vs 3.0, *p* = 0.030) and all expressed willingness to continue beyond the study (vs 50% of controls). Exploratory analyses suggested within-group improvements in pain distribution, jaw function, and pain catastrophizing in the yoga group, though between-group differences in clinical outcomes were not significant.

**Conclusion:**

This study provides valuable preliminary insights into intervention’s implementation and offers a foundation for future trials.

**Clinical Trial Registration:**

This study was retrospectively registered with Australian New Zealand Clinical Trials Registry (https://www.anzctr.org.au/) (Trial Id: ACTRN12623000069651).

## Introduction

Painful temporomandibular disorders (TMDs) are the most common non-odontogenic source of orofacial pain, with myofascial pain of the jaw muscles frequently reported [1]. Myofascial pain is a muscle pain characterised by pain on palpation of the masticatory muscles with or without limited mouth opening [2]. More complex presentations are often accompanied by psychological distress and reduced oral health–related quality of life [3, 4]. Pain is the primary reason individuals seek care, and management typically requires addressing both physical and psychosocial contributors [5, 6].

Self-management is a core component of TMD management and commonly consists of education; exercise; self-massage; thermal therapy; dietary and nutritional advice; and parafunctional behaviour identification, monitoring and avoidance [7]. Because TMDs often coexist with comorbid conditions such as neck pain and headaches, effective management needs to be multimodal and adaptable. Exercise-based approaches have shown promise: for example, an eight-week trial of therapeutic and aerobic exercise significantly improved pain intensity and bite force in TMD patients, although neuromuscular activation remained unchanged [8]. These findings highlight the potential for movement-based interventions in improving clinical outcomes. One movement-based, self-management option gaining interest is yoga.

Yoga is a mind–body practice integrating postures, breathing, and meditation. It has demonstrated benefits in several chronic pain conditions, including low back pain and neck pain. A Cochrane review indicates that yoga is more effective than not doing exercise for improving pain and back-related function, and that yoga is comparable to other types of back-focused exercise in improving function [9]. Cost effectiveness analyses have also supported yoga in patients with low back pain [10, 11].

When the present study was initiated in 2011, no trials had yet evaluated yoga for myofascial pain for jaw muscles, and evidence for TMD more broadly was sparse. Since then, a small number of studies have examined yoga-based interventions for TMD or related myofascial pain conditions including face yoga compared with dry needling [12], yoga-based daily asanas and breathing exercises [13], supervised Hatha yoga for cervical myofascial pain [14], Raj-yoga combined with conventional TMD care [15], yoga with fascial manipulation for chronic neck pain [16], and multimodal holistic approaches incorporating yoga for chronic orofacial pain [17]. Across these trials, yoga was associated with improvements in pain, mandibular or cervical function, and psychological outcomes, supporting its potential therapeutic role.

Despite these encouraging findings, important gap remains. Most trials reported excellent completion rates [12–14], but adherence was inferred rather than systematically tracked, and participants’ perceptions of yoga as a self-management strategy were seldom captured. Similarly, comparative and multimodal trials [15, 16] reported clinical outcomes but did not evaluate adherence or acceptability, while Bhalla et al. highlighted adherence challenges in holistic interventions [17]. Moreover, many studies relied primarily on patient-reported outcomes, with few testing structured yoga protocols evaluated against validated diagnostic criteria and clinician-administered assessments.

In this context, the present study represents one of the earliest attempts to evaluate a standardized yoga program for myofascial pain of masticatory muscles. It was distinctive in prospectively diagnosing participants with validated RDC/TMD criteria, incorporating clinician re-examination, and capturing detailed adherence through daily diaries alongside measures of acceptability. At the time the trial was initiated in 2011, no published yoga studies had yet addressed this condition.

Accordingly, this study was designed to examine the feasibility of implementing a structured yoga-inclusive program alongside standard care in a university population, using an active control comparator. While the original trial registration listed pain as the primary outcome, in line with current CONSORT recommendations for pilot and feasibility trials, this manuscript emphasizes feasibility - defined as adherence and acceptability - as the primary outcomes. Clinical outcomes are reported as exploratory findings intended to inform the design of a future fully powered randomized controlled trial

### Objectives

The specific objectives of this study were to evaluate feasibility and acceptability of delivering a 4-week yoga inclusive management program in the management of myofascial pain by assessing adherence rates and participants perceived acceptability, in comparison to an active control condition. The secondary objective was to explore the preliminary effects of the interventions on pain intensity, jaw function, oral health-related quality of life, and pain-related psychosocial outcomes to inform the design of a future fully powered randomized controlled trial.

## Materials and methods

### Design

**Fig. 1:**
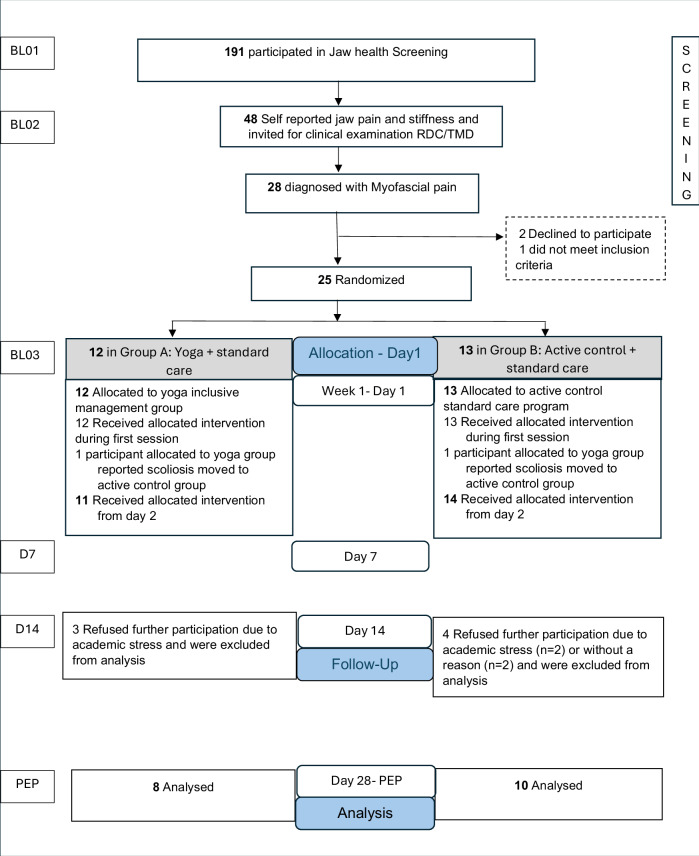
Participant flow from jaw health screening to the end of the study. BL – baseline; D7 – Day 7, D14 – Day 14, D28 – Day 28 that is Primary end point (PEP).

This was a parallel-group, open-label, single blinded randomized controlled feasibility trial evaluating a yoga-inclusive program (in addition to standard care) versus an active control (standard care plus walking and application of an unheated heat pack) for myofascial pain of masticatory muscles. Participants were recruited via a jaw-health screening procedure. The study flow and assessment timeline are shown in Fig. 1.

### Study population

Participants were dental students enrolled at the University of Sydney in 2011. Recruitment occurred between May and December 2011 across the major dentistry teaching sites in Sydney. Students received an email invitation outlining the aims and time commitment of the study, followed by a reminder email one week later. Those who agreed completed an online jaw-health screening questionnaire via a secure web link.

At the time this study was conducted, prospective registration was not routinely required for small, investigator-initiated exploratory trials. Ethical approval had been obtained in advance from the Western Sydney Local Health District Human Research Ethics Committee and The University of Sydney Human Research Ethics Committee. To meet current reporting standards and ensure transparency, the trial has since been retrospectively registered with the Australian New Zealand Clinical Trials Registry (ACTRN12623000069651). This manuscript adheres to the CONSORT extension for randomized pilot and feasibility trials, with a completed checklist provided in Supplementary Table 2 (Table S2).

### Study participant diagnosis

The Research Diagnostic Criteria for Temporomandibular Disorders **(**RDC/TMD) was used to screen and diagnose myofascial pain of the masticatory muscles in the study population. The instrument has demonstrated validity for Group Ia myofascial pain (sensitivity 0.65, specificity 0.92) and Group Ib myofascial pain with limited opening (sensitivity 0.79, specificity 0.92) [18]. The RDC/TMD assessment consisted of an online screening questionnaire completed by all study participants which includes a self-report of pain in the orofacial region, followed by a clinical examination of those reporting pain.

#### Screening procedure

The online questionnaire included the RDC/TMD patient history questions that were required to make a Myofascial pain diagnosis [2] and implemented as an electronic form (Microsoft Office excel 2003 / Survey Monkey). Participants’ de-identified responses were input directly into statistical software (SPSS version 19: SPSS Inc, Chicago, Illinois, USA).

##### Clinical evaluation

Those with self-reported orofacial pain were invited for a clinical evaluation performed by a clinician trained and calibrated in RDC/TMD assessment. Along with mobility assessments, the following muscle sites were palpated under 2 pound (2lbs/1kg) palpation pressure: the anterior temporalis, middle temporalis, posterior temporalis, masseter origin, masseter body and masseter insertion. Participants graded tenderness to palpation as follows: 0 = no pain, 1 = mild pain, 2 = moderate pain.

#### Diagnosis

To classify as myofascial pain of masticatory muscles, individuals reported pain in self-reported questionnaire and pain in at least 3 muscle sites. Individuals diagnosed with myofascial pain with or without limited mouth opening, were invited to participate in the RCT. Those with other TMDs (e.g. arthralgia, disc displacement, osteoarthritis), who were under treatment for TMDs by a health practitioner, or who had uncontrolled or debilitating cardiovascular, respiratory, musculoskeletal and neurological diseases or who were unable to attend for the entire study duration were excluded from the study. They were provided with information about standard self-care management for TMDs (as in Appendix 1) and referred to the orofacial pain clinic at Westmead Hospital for further management.

### Intervention phase

After providing informed consent, eligible participants were randomized in a 1:1 ratio to either a yoga-inclusive management program (Group A) or an active control program (Group B). Randomisation was performed independently by an individual not directly involved in the study, who drew names from a hat and sequentially allocated participants. Allocation concealment was maintained until the first intervention session. Although it was not possible to blind participants to their allocated activity, outcome assessors conducting the clinical examinations were blinded throughout. All remaining baseline assessments were collected after randomisation but prior to the start of the intervention.

Both groups received a 28-day program in addition to standard self-care education for temporomandibular disorders. Each participant attended two supervised 30 min sessions per week and was instructed to complete a daily 30 min home practice. Adherence was monitored through daily diaries, which were reviewed during supervised sessions to encourage engagement and record practice consistency.

#### Standard self-care management (Both groups)

All participants were offered Standard self-care management for myofascial pain of masticatory muscles. This was done by a discussion on self-care management strategies and by providing an email copy of ‘Immediate management of Temporomandibular disorders’ document (Appendix 1).

#### Group A - Yoga Group

Participants in Group A were taught simple yoga exercises, which included 15 min of active physical yoga exercises, 15 min of breathing exercises and meditation as detailed. The intervention was delivered in an indoor setting by a yoga instructor with experience in teaching hatha yoga and prior training in working with individuals with musculoskeletal conditions. The program followed a standardized protocol, without individual tailoring. At the first session, each participant was provided with a yoga mat and introduced to the purpose and format of the yoga program. Each 30 min session began with a brief warm-up and continued with a short sequence of 9 to 12 movements (sun salutation), followed by four sustained yoga postures, breathing exercises, and a period of focused attention or meditation. The sequence of activities was repeated over two successive sessions, with new yoga routines introduced during weeks 2, 3, and 4 to support progression. To assist with home practice, participants were emailed a copy of the relevant session routine after attending each week’s in-person session. A complete summary of the yoga intervention and progression schedule is provided in Appendix 2.

#### Group B – Active control group

Participants in Group B received an active control program designed to match the yoga group in time and researcher interaction. The program was delivered by the same instructor who facilitated group A, ensuring consistency across both arms of the trial. Activities were completed in an outdoor setting, and the intervention followed a standardized protocol for all participants. At the first session, each participant was provided with an unheated terry-towel heat pack (Head and Neck, Chattanooga Group Inc., NSW, Australia) and a SW 200 Yamax pedometer. Participants were instructed to complete a daily routine consisting of 15 min of walking in an open and clean environment, followed by 15 min of applying the unheated heat pack to the neck and masticatory muscles. During the first session, participants were trained on the use of the pedometer, including how to wear it, read the display, and reset the step counter. A three-lap walking routine was demonstrated to standardize activity. To minimize external stimulation during the heat application, participants were advised not to engage in other activities such as listening to music. Full details of the walking protocol and control routine are provided in Appendix 2.

#### Adherence monitoring

Participants recorded daily completion of their assigned activity in a diary, which was reviewed at each supervised session. This provided a continuous and verifiable measure of engagement with the intervention. Daily diary questionnaire is provided in Appendix 3.

### Outcome measures

#### Timing of assessment

The outcome assessments were conducted at baseline (BL), mid-intervention [day 7 (D7) and day 14 (D14)], and post-intervention at day 28 (primary endpoint, PEP). Baseline measures were derived from three stages: screening (BL01), clinical examination for myofascial pain diagnosis (BL02), and the remaining assessments completed after randomisation but before the first intervention session (BL03).

#### Baseline assessment

The socio-demographic characteristics, jaw pain history, and Axis I RDC/TMD data (BL01–02) were collected, alongside clinical examination findings. At BL03, participants completed a set of jaw pain outcome measures and potential confounders at the commencement of the RCT. All outcomes are described below.

To assess the course and trend of intervention on myofascial pain of jaw muscles, the outcome measures were obtained on day 7, day 14 and day 28 (PEP) (Supplementary table – Table S1).

#### Primary outcomes

##### Feasibility (adherence rate)

Feasibility was evaluated from daily practice adherence. Adherence was defined as the proportion of the 28 prescribed days on which the assigned activity was completed as instructed. Adherence data were obtained from participant-completed daily diaries and verified at each supervised session. This provided a continuous measure of adherence, expressed as the proportion of prescribed days completed. Although no adherence threshold was pre-specified in the trial protocol, for interpretation we used a literature-informed benchmark of ≥75% adherence as an exploratory reference, consistent with published RCTs of yoga for chronic pain, where ≥70–75% class attendance and/or ≥3 days per week home practice are commonly used as benchmarks of adequate exposure [19, 20]. Using this criterion allows comparison with previous studies and reflects a dose likely sufficient to achieve therapeutic benefit.

##### Acceptability

Quantitative measure: At the end of the 28-day intervention, participants completed a post-study questionnaire rating the perceived helpfulness or harmfulness of their assigned intervention on a scale from –10 (extremely harmful) to +10 (extremely helpful), with 0 indicating neither helpful nor harmful. This rating provided a direct quantitative measure of participant-perceived acceptability.

Qualitative measures: At the same time point, participants were asked whether they would be willing to continue the assigned activity beyond the study period. They were also asked to guess which group (active or control) they believed they had been assigned to. These responses provided additional insights into acceptability and the credibility of the blinding.

#### Secondary outcomes

##### Key exploratory clinical outcomes

Pain intensity and distribution (Self report): Pain intensity was measured using the Characteristic Pain Intensity (CPI; 0–100) subscale of the Graded Chronic Pain Scale. For this study, the standard 6-month reference period was reduced to 7 days for the day-7 and day-14 assessments, and to 2 weeks for the day-28 assessment. For interpretation, published recommendations for TMD and chronic pain suggests a ≥30% relative reduction, in CPI represents a minimal clinically important difference (MCID) [21, 22]. This threshold was noted as a reference for future trials, although no formal responder analysis was conducted in the present feasibility study.

**Fig. 2:**
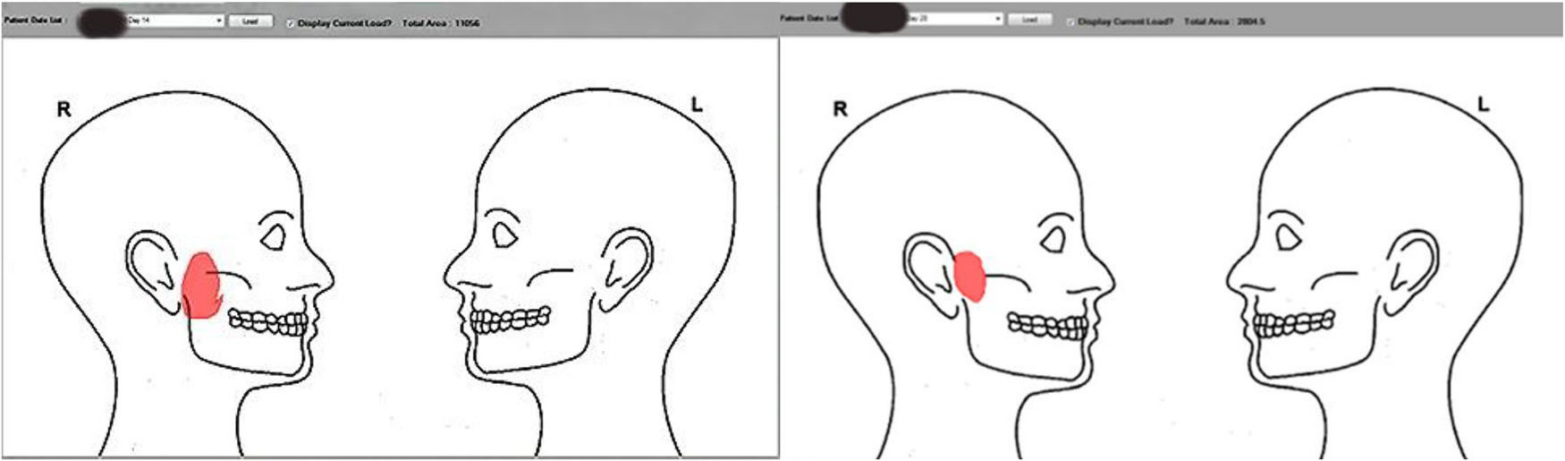
In this example, a patient marked painful regions on day 14 and again on day 28 using the digital pain map of the head and neck.

Pain location and distribution were captured with a digital pain map of the head and neck region (Fig. 2). Participants marked pain areas on standardized lateral and frontal head-and-neck outlines using a computer cursor. Each marked area was encrypted and transmitted to a network device that quantified the mapped region’s pixels as a measure of pain area.

Jaw function (Self report): The Jaw Functional Limitation Scale (JFLS-20) was used to assess functional status of the masticatory system across three domains: mastication, jaw mobility, and verbal/emotional expression [23].

Clinical examination: RDC/TMD Axis I data were collected at baseline and day 28. Muscle palpation at six sites [temporalis (anterior, middle, posterior); masseter (origin, body, insertion)] was performed by a calibrated, blinded clinician. Tenderness scores were summed to create a total palpation score, representing overall masticatory muscle tenderness. This approach has been used in prior TMD research to quantify muscle pain burden more comprehensively [24]. Note that this clinician was blinded to group assignment of each participant.

##### Other exploratory outcomes

Oral Health related Quality of Life: The Oral Health Impact Profile – 14 (OHIP 14) assesses oral health related quality of life across 7 sub-scales of functional limitation, physical pain, psychological discomfort, physical and psychological disabilities and handicap [25].

Psychosocial outcomes: Psychosocial impairment was assessed with three instruments; the Pain Catastrophizing Scale (PCS) to assess catastrophic thinking related to pain [26], the Depression, Anxiety and Stress Scale (DASS-21) [27], and the pain self-efficacy questionnaire (PSEQ) [28] to assess the confidence, in people with pain, to perform a range of activities including household chores, socialising, and coping with pain without medications.

### Calculating assessment values

For each secondary outcome measure, change scores were calculated as the difference between baseline and end-of-intervention (day 28) values. For pain map, participant marked pain regions at each assessment point (BL, D7, D14, PEP). The change is pain map is the difference between the total marked area (in pixels) on day 28 and the total area at baseline. These change scores were used in between-group analyses.

### Data analysis

#### Sample size determination

As this was a development and feasibility study, a formal sample size calculation was not performed. A total of 20 participants (10 per group) was targeted, in line with recommendations for pilot and feasibility trials [29].

#### Testing for normality

Distributional properties of continuous outcomes were examined visually (histograms) and formally tested using the Shapiro–Wilk test.

#### Baseline characteristics

Demographic and clinical variables were summarized descriptively as mean (SD) for continuous variables and (n) for categorical variables.

#### Primary outcomes

Feasibility (adherence rate) and acceptability (perceived helpfulness score) were normally distributed and are presented as mean with 95% confidence intervals. Between-group differences were assessed using independent-samples *t* tests. Categorical acceptability outcomes (willingness to continue, group guess) were summarized as proportions and compared using chi-square tests.

#### Secondary outcomes

For exploratory clinical measures (CPI, JFLS-20, OHIP-14, PCS, DASS-21, PSEQ, palpation tenderness), change scores were analyzed. Because the distribution of change scores was not normally distributed and the sample size was small, non-parametric Mann–Whitney *U* tests were used to compare groups. Results are reported as medians and interquartile ranges (IQRs). In addition, within-subject changes across time points (BL, D7, D14, PEP) were examined using the Friedman test, with post hoc Wilcoxon signed-rank tests applied where appropriate.

All analyses followed a per-protocol approach. Statistical significance was set at *p* < 0.05 (two-tailed). Analyses were performed using IBM SPSS statistics for Windows (version 29).

## Results

### Participant flow and recruitment

A total of 262 students were emailed an invitation to complete the online jaw health screening, and 191 (73% response rate) completed the questionnaires (90 male, 101 female; mean age 23.9 years, SD 3.3). Non-response was due to the voluntary nature of the screening and its timing during periods of academic commitments at the University. Of those, who responded, 48 reported jaw and orofacial pain and were invited for clinical examination. Twenty-eight students (14.6% of all respondents; 58% of those self-reporting pain diagnosed with myofascial pain of the masticatory muscles according to RDC/TMD criteria and were invited to participate in the RCT.

Of the 28 eligible individuals, 25 provided informed consent and were randomized to either the yoga-inclusive management program (*n *= 12; 8 female) or the active control standard care group (*n *= 13; 10 female). During the first supervised yoga session, one female participant reported scoliosis and was reassigned to the control group because the standardized yoga program could not be modified for her condition. Three participants in the yoga group withdrew (reason: academic stress and lack of time), and four participants in the control group withdrew (reasons: academic stress/lack of time, *n* = 2; no reason provided, *n *= 2) prior to completing the study. This resulted in a final analytic sample of eight participants in the yoga group and ten in the active control group. A participant flow diagram is presented in Fig. 1.

### Baseline information

The demographic details and baseline scores of participants are summarised in Table 1. The two groups were broadly similar in age and gender. Baseline clinical measures, including pain intensity, jaw function, oral health–related quality of life, psychosocial measures, and palpation tenderness, were also comparable across groups. Most participants reported low pain intensity at baseline; only two participants in the yoga group reported high pain intensity. Both groups demonstrated mean pain self-efficacy scores above 40, reflecting minimal impairment in confidence to perform daily activities despite pain.

**Table 1:** Baseline characteristics of participants in active control and yoga group.

Domain	Outcome measures	Active Control (*N* = 10)	Yoga (*N *= 8)
Sociodemographic	Age in years (m,SD)	23.8 (4.5)	23.7 (2.7)
	Sex, Female: Male (*n*)	8:2	4:4
	Pain duration in months (m,SD)	11.7 (22.5)	17.7 (23.9)
	Medication for facial pain (*n*)	0	1
	Medication for mental health (*n*)	0	1
	Exercise/physical activity (*n*/total *n*)	2 / 6	1 / 3
Pain	CPI^a^ (m,SD)	22.3 (19.9)	29.1 (27.7)
	Pain map^a^ (m,SD)	7579.5 (6622.1)	12773.1 (10556.6)
	Pain Disability^a^ (m,SD)	0 (0)	0.1 (0.3)
Jaw Function	JFLS^a^ (m, SD)	15.9 (21.8)	16.0 (26.4)
Oral Health QoL	OHIP-14^a^ (m, SD)	11.6 (7.0)	11.7 (8.6)
Psychosocial factors	DASS stress^a^ (m, SD)	8.4 (6.9)	9.0 (9.7)
	DASS anxiety^a^ (m, SD)	8.0 (8.9)	7.0 (7.7)
	DASS depression^a^ (m, SD)	7.8 (7.3)	9.7 (10.3)
	PCS^a^ (m, SD)	9.5 (9.8)	19.2 (11.7)
	PSEQ^b^ (m, SD)	55.0 (7.1)	46.7 (8.3)
Clinical parameters	Pain-free opening (m, SD)	44.2 (6.0)	47.9 (10.6)
	Max unassisted opening (m, SD)	51.4 (4.1)	57.6 (8.4)
	Max assisted opening (m, SD)	54.1 (3.9)	62.4 (8.1)
	Total palpation score^a^ (m, SD)	5.2 (2.8)	6.8 (4.2)

*m*, *SD* mean, standard deviation; *N* number. Positive responses to potential confounders are reported, with the exercise/physical activity item answered by 9 participants (Control = 6; Yoga = 3). *CPI* Characteristic Pain Intensity, *JFLS* Jaw Functional Limitation Scale, *OHQoL* Oral Health Quality of Life, *OHIP*Oral Health Impact Profile, *DASS* Depression, Anxiety and Stress Scale, *PSEQ* Pain Self-Efficacy Questionnaire, *PCS* Pain Catastrophising Scale.

^a^Lower values indicate better status.

^b^Higher values indicate better status.

At the primary endpoint (Day 28), several outcome measures significantly deviated from normality according to the Shapiro–Wilk test, including characteristic pain intensity (Control: *p* = 0.004; Yoga: *p* = 0.005), total pain mapping area (Control: *p* < 0.001; Yoga: *p* < 0.001), total disability points (Yoga: *p* < 0.001), jaw function limitation score (Control: *p* = 0.002; Yoga: *p* < 0.001), DASS anxiety (Yoga: *p* = 0.027), DASS depression (Control: *p* = 0.015), OHIP (Control: *p* = 0.003; Yoga: *p* < 0.001), pain catastrophizing score (Control: *p* = 0.025), and pain self-efficacy score (Control and Yoga: *p* < 0.001). As a result, nonparametric tests (Mann–Whitney U) for between-group comparisons and Friedman test for within-group changes across time points for the final analysis. By contrast, the primary outcome measures of adherence and post-study acceptability were normally distributed, and these were analyzed using independent-samples *t* tests for continuous variables and chi-square tests for categorical variables.

### Primary outcomes

#### Feasibility (adherence)

All participants attended the seven supervised sessions. The average adherence to the 28-day intervention, based on daily diaries, was 24 days (85%) in the yoga group and 19 days (68%) in the active control group. This difference was statistically significant (*p* = 0.048, independent *t*-test), indicating greater adherence in the yoga group (Table 2).

**Table 2:** Changes in outcome measure 28 days post intervention in active control and yoga group.

Domain	Outcome measures	Active Control *N* = 10	Yoga *N* = 8	*p*-value
Feasibility^1^ &	Days intervention performed (m, 95% CI)	19 [14–23]	24 [21–27]	**0.048**_a_*
Acceptability^1^	Intervention benefit/harm (−10 to 10) scale (m, 95% CI)	3 [1–5]	6.3 [4–8]	**0.03**_a_*
Pain^2^	CPI (PEP–BL)	♦ 1.7 (−13.3 to 7.5)	♦ −1.7 (−34.2 to 8.3)	0.515
Pain^2^	Facial pain mapping area (PEP–BL)	♦ −1447.5 (−10368.0 to 951.8)	♦ −13264.8 (−16804.1 to −1393.5)	0.122
Pain^2^	Pain-related disability (PEP–BL)	♦ 0.0	♦ 0.0	0.696
Jaw Function^2^	JFLS-20 (PEP–BL)	♦ −1.5 (−11.0 to 1.0)	♦ −3.5 (−14.3 to −0.8)	0.460
OHQoL^2^	OHIP-14 (PEP–BL)	♦ −2.0 (−6.5 to −2.0)	♦ −7.5 (−11.0 to −0.3)	0.633
Psychosocial factor^b^	DASS stress (PEP–BL)	♦ −2.0 (−6.5 to 0.5)	♦ −2.0 (−8.5 to 3.0)	0.829
Psychosocial factor^2^	DASS anxiety (PEP–BL)	♦ −2.0 (−8.0 to 0.0)	♦ −3.0 (−7.0 to 9.0)	0.633
Psychosocial factor^2^	DASS depression (PEP–BL)	♦ −5.0 (−8.0 to 0)	♦ 1.0 (−13.5 to 5)	0.237
Psychosocial factor^2^	PCS (PEP–BL)	♦ −2.0 (−5.8 to 0.0)	♦ −8.5 (−12.0 to −3.5)	0.027*
Psychosocial factor^2^	PSEQ (PEP–BL)	♦ 0.0 (−2.8 to 3.0)	♦ 11.0 (3.3 to 15.8)	0.003*
Clinical parameter^2^	Pain free opening (PEP–BL)	♦ 2.0 (−3.5 to 6.3)	♦ 7.0 (1.0 to 19.3)	0.101
Clinical parameter^2^	Maximum unassisted opening (PEP–BL)	♦ −0.5 (−2.5 to 1.5)	♦ 2.0 (0.6 to 7.3)	0.083
Clinical parameter^2^	Maximum assisted opening (PEP–BL)	♦ 0.0 (−1.8 to 2.0)	♦ 0.3 (−2.0 to 2.5)	0.762
Clinical parameter^2^	Total palpation score (PEP–BL)	♦ −3.0 (−4.0 to 1.0)	♦ −2.0 (−5.5 to 0.8)	0.762

Outcomes with normally distributed data are reported as mean (95% CI) and compared using independent *t*-tests (a). Outcomes with non-normally distributed data are reported as ♦ median (interquartile range) and compared using Mann–Whitney *U* tests. Statistically significant results (*p* < 0.05) are shown in bold with an *asterisk. Primary End Point (PEP) is 28 days post-intervention commencement*, BL* Baseline, *CPI*Characteristic Pain Intensity, *PSEQ* Pain Self-Efficacy Questionnaire, *PCS* Pain Catastrophising Scale, *JFLS* Jaw Functional Limitation Scale, *OHQoL* Oral Health Quality of Life, *OHIP* Oral Health Impact Profile, *DASS* Depression, Anxiety and Stress Scale. ^1^primary outcome, ^2^secondary outcome.

#### Acceptability

Participants in the yoga group reported higher perceived benefit (mean = 6.3, SD = 2.3) than those in the active control group (mean = 3.0, SD = 3.4). The independent *t*-test confirmed a statistically significant difference between groups (*p* = 0.030) (Table 2).

### Intention to continue the intervention

**Fig. 3:**
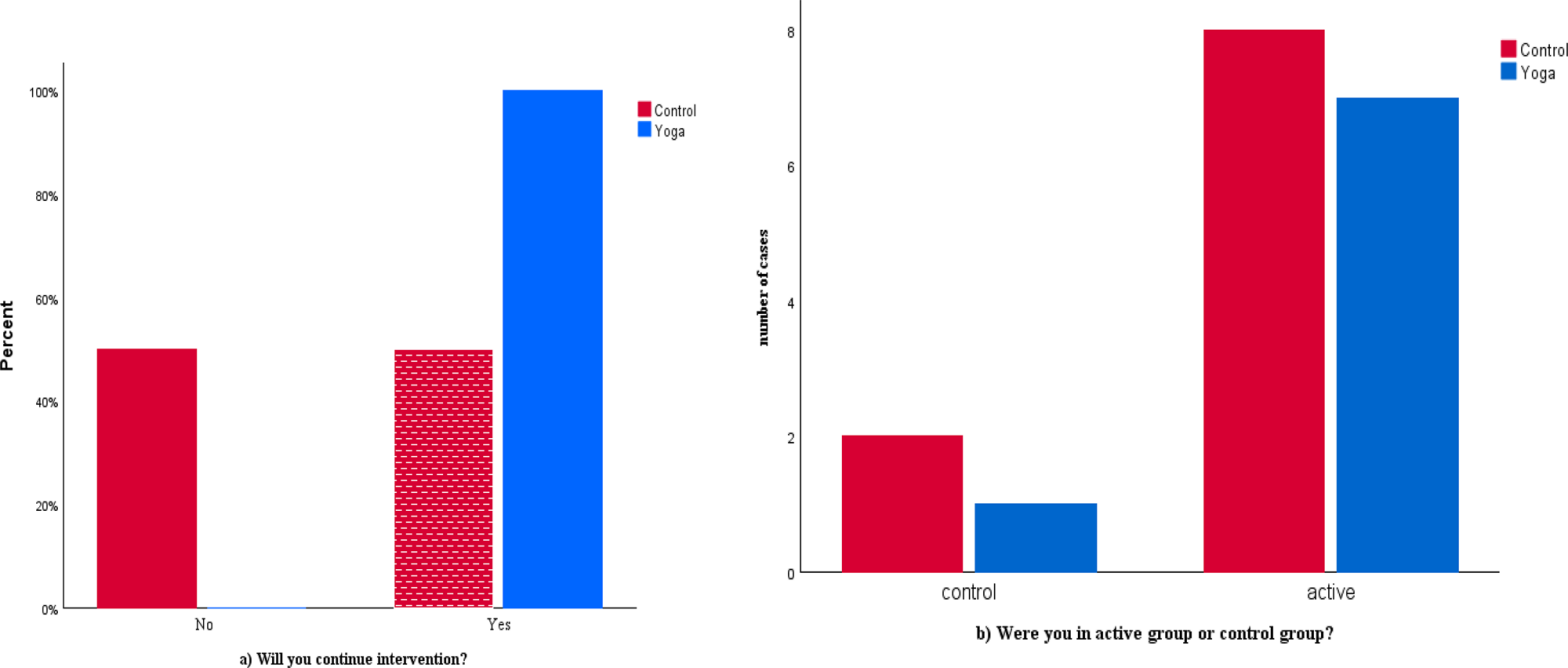
Participants qualitative measures of acceptability. **a** Participants’ willingness to continue intervention, and **b** participants’ belief in the intervention.

There was a significant difference between groups in participants’ intention to continue the assigned activity after the 28-day intervention (*p* = 0.035, chi-square test) [Fig. 3a]. All yoga participants (100%) expressed willingness to continue, compared to only 50% in the active control group.

### Perceptions of group allocation

Eight participants (80%) in the active control group and seven (87.5%) in the yoga group believed they were in the “active group.” Conversely, two participants (20%) in the active control group and one (12.5%) in the yoga group believed they were in the “control group” [Fig. 3b].

### Secondary outcomes

#### Secondary key exploratory outcomes (pain and jaw function: self-report & clinical examination)

**Fig. 4:**
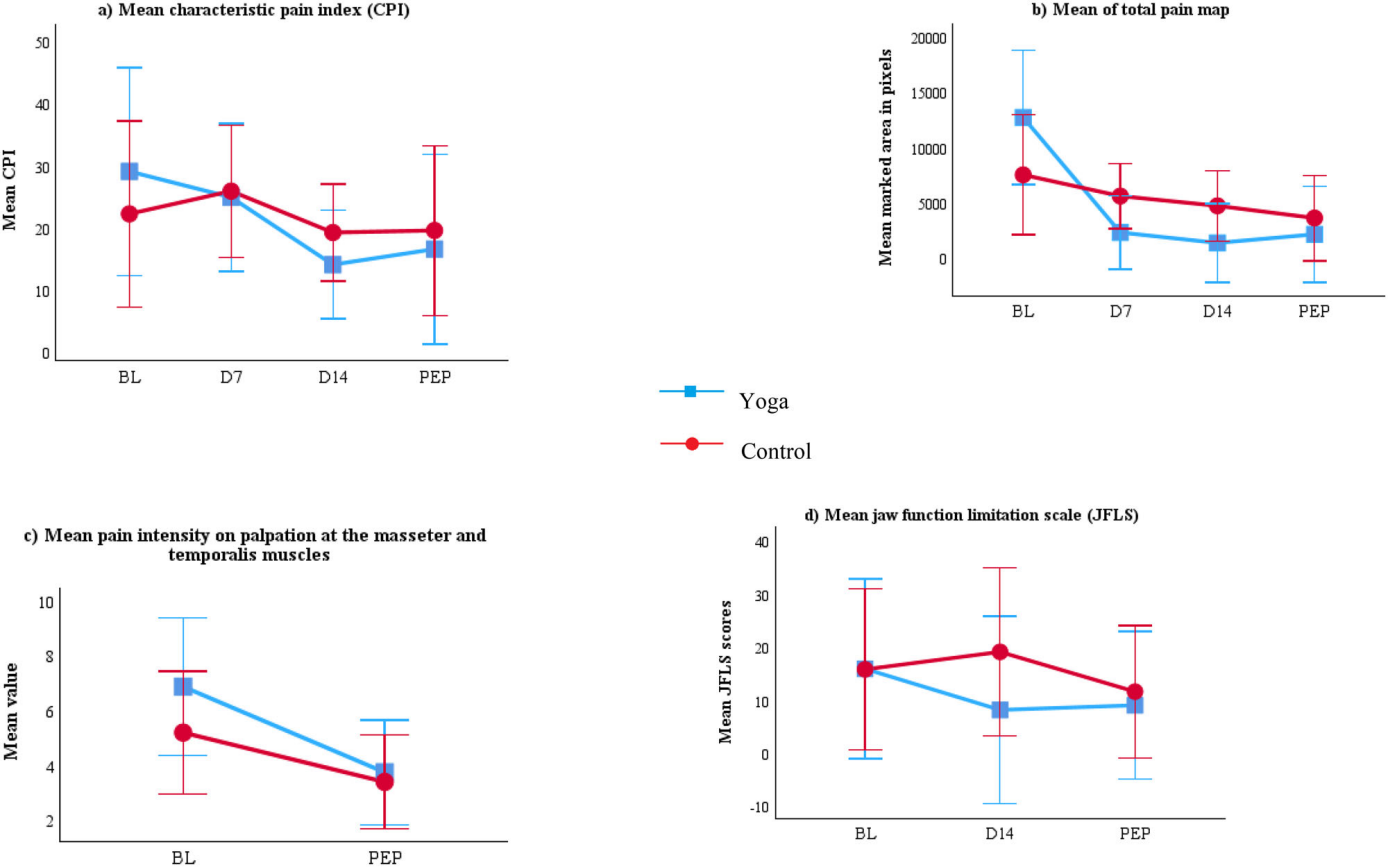
Mean and standard error of self-reported pain outcomes (3a, 3b), clinical pain outcome (3c), and self-reported jaw function measures (3 d) at baseline (BL), day 7 (D7), day 14 (D14), and primary endpoint (PEP, day 28).

The magnitude of change in characteristic pain intensity, pain distribution, and jaw function between baseline and the primary endpoint (Day 28) did not differ significantly between the yoga and control groups (Mann–Whitney U test) (Table 2, Fig. 4). However, within-group analyses indicated changes in the yoga group. Pain distribution, as measured by the digital pain map, showed a significant effect of time (Friedman χ²(df), p): (χ² (3) = 12.11, *p *= 0.007), with post hoc Wilcoxon signed-rank tests showing reductions at day 7, day 14, and day 28 compared with baseline. Jaw function (JFLS-20) also showed a significant effect of time in the yoga group (χ² (3) = 10.19, *p* = 0.006), with post hoc tests indicating improvements at day 14 and day 28 compared with baseline. No significant within-group effects were observed in the control group.

For clinical parameters, maximum unassisted jaw opening improved significantly within the yoga group (*χ²* (1) = 4.5, *p* = 0.034). However, these changes were small and not considered clinically meaningful, as both groups demonstrated values within the normal functional range (>50 mm).

#### Other secondary exploratory outcomes (OHQoL psychosocial measures)

Between-group analysis using the Mann–Whitney test showed no significant differences between groups for most outcomes. The only exceptions were pain catastrophizing (PCS; *p* = 0.027) and pain self-efficacy (PSEQ; *p* = 0.003), where greater improvements were observed in the yoga group (Table 2). Within-group analyses further indicated that PCS showed a significant effect of time in the yoga group (χ²(2) = 7.16, p = 0.028), with post hoc Wilcoxon signed-rank tests showing reductions from post-intervention compared with baseline. PSEQ also showed a significant effect of time (χ²(2) = 10.06, *p* = 0.007), with post hoc tests demonstrating improvements from post-intervention compared with baseline and between day 14 and baseline.

**Fig. 5:**
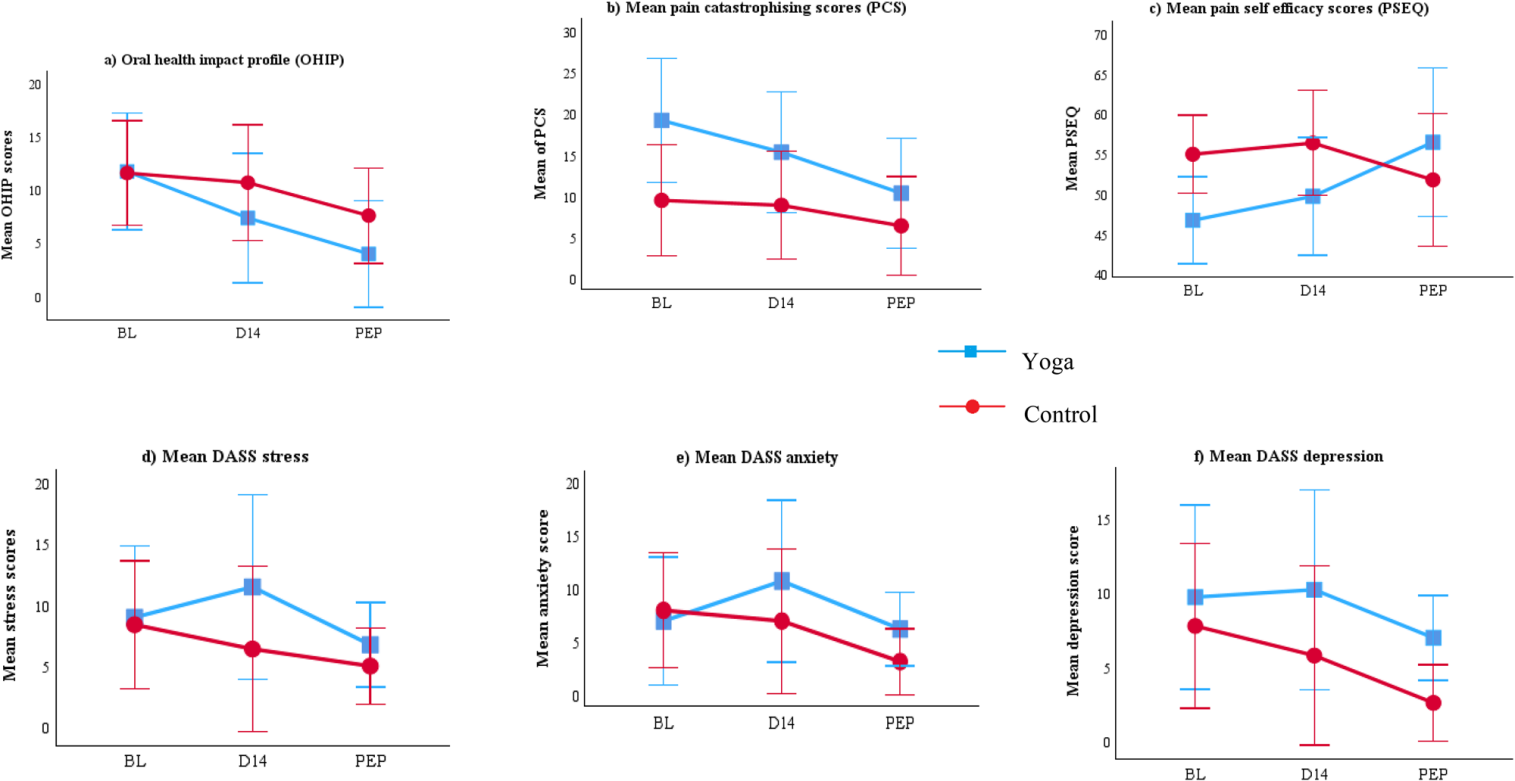
Mean and standard error of secondary exploratory outcomes at baseline (BL), day 14 (D14), and primary endpoint (PEP, day 28). The figure illustrates the trend over time for **a** OHIP; **b** PCS **c** PSEQ **d**–**f** DASS stress, anxiety and depression. Higher pain self-efficacy scores indicate better status, while lower values indicate better outcomes for other parameters. DASS Depression, Anxiety, and Stress Scale.

Oral health impact profile (OHIP-14) demonstrated significant within-group effects in both groups (control: χ² (2) = 13.47, *p* = 0.001; yoga: χ² (2) = 8.24, *p* = 0.016) Fig. 5. Post hoc analysis indicated improvements at post-intervention compared with baseline and at day 14 compared with baseline in both groups.

Within the control group, depression scores (DASS-21 subscale) showed a significant effect of time (χ²(2) = 6.66, *p* = 0.036), with post hoc tests confirming reductions from post-intervention compared with baseline. No significant changes were observed for anxiety, stress, or palpation tenderness in either group.

## Discussion

This feasibility study evaluated a yoga-inclusive management program for myofascial pain of masticatory muscles compared with an active control. Adherence was higher in the yoga group (85%) than in controls (68%), meeting the ≥75% benchmark commonly used in yoga RCTs [19, 20]. Participants also reported greater perceived benefit and stronger willingness to continue yoga, supporting its acceptability. These findings align with the small but growing literature suggesting yoga is both feasible and engaging for patients with orofacial and myofascial pain [12, 13, 15].

Several limitations must be acknowledged upfront. The trial was retrospectively registered and there was a considerable delay between data collection and reporting, reflecting practical constraints such as participant graduation and investigator relocation. The standards for feasibility and pilot studies have since evolved, and the study should be interpreted within that context.

The study was small (*n* = 18), underpowered to detect between-group effects, and included dental students who were not treatment-seeking, reported mild pain, and were familiar with TMD management through their training. This likely limited generalisability and reduced sensitivity to detect clinically meaningful change. Retention was a challenge, with 28% dropout, largely due to academic commitments. Some baseline measures were collected after randomisation, and interventions differed in environment (yoga indoors vs walking outdoors), which may have influenced experience and outcomes. Low baseline pain may have produced a floor effect, and acute vs chronic cases were not distinguished. The trial also lacked a non-active comparator, follow-up beyond four weeks, and data on sleep or comorbidities. These limitations should inform refinement of future trial design.

### Exploratory clinical outcomes: pain and function

Although the study was not powered to detect efficacy, exploratory analyses suggested that yoga participants experienced within-group improvements in pain distribution and jaw function. These findings are broadly consistent with prior exercise-based interventions for TMD. For instance, Moleirinho-Alves et al. [8] demonstrated that an eight-week program of therapeutic and aerobic exercise reduced masticatory muscle pain and improved bite force, while yoga-based interventions in TMD and cervical myofascial pain have shown improvements in mandibular mobility and pain reduction ([13, 14]. The present study adds to this evidence by applying validated RDC/TMD re-examination at follow-up, strengthening the objectivity of the observed changes. Future definitive trials should incorporate clinically meaningful thresholds, such as the MCID of 20–30 points or ≥30% reduction in CPI, to enhance interpretability and patient relevance of findings [21, 22].

### Other exploratory outcomes

Exploratory analyses also suggested possible improvements in oral health–related quality of life and cognitive–emotional measures. The yoga group showed reduced catastrophizing (PCS, *p* = 0.027) and improved pain self-efficacy (PSEQ, *p* = 0.003) compared with controls, though the latter may partly reflect regression to the mean given lower baseline scores. In contrast, depression, anxiety, and stress (DASS-21) improved more in the active control group than in yoga, contrary to prior TMD/myofascial yoga studies that reported psychosocial benefits [13, 15, 17]. Given the low baseline psychological distress in this cohort and the inclusion of walking in the control condition—which is independently associated with psychological wellbeing and mood regulation [30, 31]. This highlights the value—and the challenge—of using an active control: both interventions may have conferred genuine benefit, albeit through different mechanisms. Importantly, participants in the yoga group still rated their program as more beneficial and were more motivated to continue, underscoring its acceptability even when comparator activities were also credible.

### How Might Yoga Work?

Yoga is more than stretching. It integrates postures, breath regulation, and mindfulness, creating a practice that engages both body and mind. Physically, yoga may relieve muscle tension, improve posture, and increase mobility in the jaw and cervical regions. Psychologically, mindfulness and breath awareness can enhance pain inhibition and support cognitive reappraisal, processes linked with reduced pain perception and distress [32, 33]. Over time, sustained practice may even promote neuroplastic changes that shift the nervous system toward parasympathetic dominance and positive affective states [34]. While these mechanisms likely require longer engagement than the four weeks tested here, even this short program was feasible and well-received, providing a platform for deeper investigation.

### Comparator considerations

The active control - walking combined with application of an unheated towel was designed to control for time, attention, and expectancy. Yet, as the psychological outcomes suggest, walking is itself an effective health-promoting activity. This raises important questions about comparator choice. Designing sham interventions for yoga is inherently challenging, as postures, breath, and meditation are difficult to mimic without introducing therapeutic elements. Nonetheless, the use of a credible, active control remains a strength, ensuring that findings are not simply due to nonspecific attention effects. The absence of between-group differences in this feasibility trial highlights the need for a larger, well-powered study to disentangle yoga-specific effects from those of comparators.

Preliminary sample size estimation from the present study suggests that a future RCT would require approximately 146 participants (73 per group) to achieve 80% power at a two-tailed 5% significance level. This calculation was based on the observed mean change in CPI (2.7 vs 12.5 between groups) and a pooled standard deviation of 21. Allowing for attrition, the target enrolment should be increased (e.g., to ~180 participants for 20% attrition), consistent with the withdrawal rate observed in this feasibility trial.

### Strengths and implications for future research

The strength of this study lies in its study design and methodology. The design of painful TMD screening streamlined participants to the study’s requirements. The use of online questionnaires and multidimensional outcome measures via objective and subjective tests also strengthen the methodology of the study. The inclusion of an active control allowed for a more rigorous test of feasibility. Importantly, participants were formally diagnosed using RDC/TMD, and re-examined at the end of trial ensuring that outcomes were grounded in validated diagnostic criteria.

A fully powered RCT is now warranted. Such a trial should recruit from treatment-seeking populations with greater symptom burden, extend the duration and follow-up to capture longer-term effects, and include broader outcome measures such as sleep and comorbidities. Comparator design will be crucial, balancing credibility with the need to isolate yoga-specific effects. Finally, cost-effectiveness should be explored. Evidence from low back pain suggests yoga can reduce healthcare utilization and improve productivity at relatively low cost [11, 35]. If similar findings hold for TMD, yoga could offer not only a clinically meaningful but also an economically sustainable management option.

## Conclusion

This feasibility study shows that a yoga-inclusive program for myofascial pain of masticatory muscles can be delivered, is acceptable to participants, and achieves adherence rates above established benchmarks. While exploratory outcomes were limited by sample size and baseline characteristics, trends in pain, function, and psychosocial measures mirror those seen in other yoga and exercise studies. With methodological refinements and adequate power, a future RCT could rigorously test the effectiveness and cost-effectiveness of yoga as an adjunctive self-management strategy for temporomandibular disorders.

## Supplementary information

Supplementary Table 1 (Table S1); Supplementary Table 2 (Table S2)

Appendix 1

Appendix 2

Appendix 3

## Data Availability

The data available on request from the corresponding author.
