# Supplementary material for: Yoga for myofascial pain of masticatory muscles – a development and feasibility study
Source: BDJ Open. 2025 Dec 10;11:94. doi: 10.1038/s41405-025-00377-x (PMC12696018; doi:10.1038/s41405-025-00377-x)
Supplement: Supplementary file 1 — Supplementary Table 1 (Table S1); Supplementary Table 2 (Table S2) [file 41405_2025_377_MOESM1_ESM.docx]

**Supplementary table 1 Baseline and outcome data acquisition timepoints**

| *Domain* | *Measures* | *BL* | *D 7* | *D 14* | *Day 28 /(PEP)* |
| --- | --- | --- | --- | --- | --- |
| Feasibility^1^ & | Daily diary – Adherence rate (completed everyday) | ✔ | ✔ | ✔ | ✔ |
| Acceptability^1^ | Participant perceived acceptability/Intervention helpfulness |  |  |  | ✔ |
|  | Post study participant behaviour/attitude |  |  |  | ✔ |
|  |  |  |  |  |  |
| Baseline | Sociodemographic characteristics | ✔ |  |  |  |
| Information | Jaw pain history | ✔ |  |  |  |
|  | Axis I RDC TMD | ✔ |  |  |  |
| Pain | Characteristic Pain Intensity (CPI) | ✔ | ✔ | ✔ | ✔ |
|  | Digitized pain mapping of head and neck | ✔ | ✔ | ✔ | ✔ |
|  | Pain related disability using Graded Chronic Pain Scale (GCPS) | ✔ | ✔ | ✔ | ✔ |
| Jaw Function | Jaw Function Limitation Scale (JFLS) | ✔ |  | ✔ | ✔ |
| Oral Health Quality of Life (OHQoL) | Oral Health Impact Profile-14 (OHIP-14) | ✔ |  | ✔ | ✔ |
| Cognitive Factors | Depression, Anxiety and Stress Scale (DASS) | ✔ |  | ✔ | ✔ |
|  | Pain Catastrophizing Scale (PCS) | ✔ |  | ✔ | ✔ |
|  | Pain Self-Efficacy Questionnaire (PSEQ) | ✔ |  | ✔ | ✔ |
| Clinical Parameters | Axis I RDC TMD | ✔ |  |  | ✔ |
| Potential Confounders | Use of medication  Exercise and Physical activity | ✔  ✔ |  |  |  |
|  |  |  |  |  |  |

Note: ^1^ = primary outcome. BL = Baseline; D7 = Day 7; D14 = Day 14; PEP is Primary End Point is 28 days post-intervention commencement

**Supplementary table 2: Consort checklist for randomised pilot and feasibility trials**

| **CONSORT CHECKLIST** | | | |  |
| --- | --- | --- | --- | --- |
|  |  |  |  |  |
| **Section/Topic** | **Item No** | **Extension for Pilot checklist** | **Reported on Page No (Pg)** |  |
| **Title and abstract** | | | |  |
|  | 1a | Identification as a pilot or feasibility randomised trial in the title | Pg 1 (Title) not stated as randomised |  |
|  | 1b | Structured summary of pilot trial design, methods, results, and conclusions (for specific guidance see CONSORT abstract extension for pilot trials | Pg 2 (Abstract) |  |
| **Introduction** | | | |  |
| Background and objectives | 2a | Scientific background and explanation of rationale for future definitive trial, and reasons for randomised pilot trial | Pg 5,6 |  |
|  | 2b | Specific objectives or research questions for pilot trial | Pg 7 |  |
| **Methods** | | | |  |
| Trial design | 3a | Description of pilot trial design (such as parallel, factorial) including allocation ratio | Pg 7 (2.1 Design) |  |
|  | 3b | Important changes to methods after pilot trial commencement (such as eligibility criteria), with reasons | Pg 8 (ethics & retrospective registration); Pg 22 (Discussion: limitations) |  |
| Participants | 4a | Eligibility criteria | Pg 9 (2.3 Diagnosis) |  |
|  | 4b |  |  |  |
|  | 4c | How participants were identified and consented | Pg 8,9 (Recruitment & consent) |  |
| Interventions | 5 | Full details of intervention | Pg 10-12, appendix 2 |  |
| Outcomes | 6a | Completely defined prespecified assessments or measurements to address each pilot trial objective specified in 2b, including how and when they were assessed | Pg 12-15 (2.5 Outcomes); Table S1 |  |
|  | 6b | Any changes to pilot trial assessments or measurements after the pilot trial commenced, with reasons | No |  |
|  | 6c | If applicable, prespecified criteria used to judge whether, or how, to proceed with future definitive trial | Exploratory reference to ≥75% adherence benchmark Pg 11–12 |  |
| Sample size | 7a | Rationale for numbers in the pilot trial | Pg 16 (2.7 Data analysis – sample size) |  |
|  | 7b |  |  |  |
| Randomisation: |  |  |  |  |
| Sequence generation | 8a | Methods used | Pg 10 (2.4 Intervention phase) |  |
|  | 8b | Type of randomisation(s); details of any restriction (such as blocking and block size) | Pg 10 (simple ratio 1:1) |  |
| Allocation concealment mechanism | 9 | Method of concealment | Pg 10 (2.4 Intervention phase) |  |
| Implementation | 10 |  |  |  |
| Blinding | 11a | Blinding (who was blinded) | Pg 10 |  |
|  | 11b |  |  |  |
| Statistical methods | 12a | Methods used to address each pilot trial objective whether qualitative or quantitative | Pg 13-17 |  |
|  | 12b | Not applicable |  |  |
| **Results** | | | |  |
| Participant flow (a diagram is strongly recommended) | 13a | For each group, the numbers of participants who were approached and/or assessed for eligibility, randomly assigned, received intended treatment, and were assessed for each objective | Mentioned in the figure1 |  |
|  | 13b |  |  |  |
| Recruitment | 14a |  |  |  |
|  | 14b | Why the pilot trial ended or was stopped | Not applicable |  |
| Baseline data | 15 |  |  |  |
| Numbers analysed | 16 | For each objective, number of participants (denominator) included in each analysis. If relevant, these numbers should be by randomised group | change in number after first intervention session is mentioned, fig 1, Tabel1 |  |
| Outcomes and estimation | 17a | For each objective, results including expressions of uncertainty (such as 95% confidence interval) for any estimates. If relevant, these results should be by randomised group | Pg 31, Table 2 |  |
|  | 17b | Not applicable |  |  |
| Ancillary analyses | 18 | Results of any other analyses performed that could be used to inform the future definitive trial | Preliminary sample size Pg 24,25 |  |
| Harms | 19 |  |  |  |
|  | 19a | If relevant, other important unintended consequences |  |  |
| **Discussion** | | | |  |
| Limitations | 20 | Pilot trial limitations, addressing sources of potential bias and remaining uncertainty about feasibility | Pg 22 |  |
| Generalisability | 21 | Generalisability (applicability) of pilot trial methods and findings to future definitive trial and other studies | Pg 21, 25 |  |
| Interpretation | 22 | Interpretation consistent with pilot trial objectives and findings, balancing potential benefits and harms, and considering other relevant evidence | Pg 21-25 |  |
| **Other information** | |  |  |  |
| Registration | 23 | Registration number for pilot trial and name of trial registry | Pg 3,8 |  |
| Protocol | 24 | Where the pilot trial protocol can be accessed, if available | Pg 3,8 |  |
| Funding | 25 |  |  |  |
|  |  | Ethical approval or approval by research review committee, confirmed with reference number | Pg 3,8 |  |
|  |  |  |  |  |
|  |  |  |  |  |
