# Supplementary material for: Yoga for myofascial pain of masticatory muscles – a development and feasibility study
Source: BDJ Open. 2025 Dec 10;11:94. doi: 10.1038/s41405-025-00377-x (PMC12696018; doi:10.1038/s41405-025-00377-x)
Supplement: Supplementary file 2 — Appendix 1 [file 41405_2025_377_MOESM2_ESM.pdf]

Appendix 1: Standard care management

The following self-care management information for temporomandibular disorders was provided to all participants during the initial supervisory session.

THE UNIVERSITY OF SYDNEY

Faculty of Dentistry

Immediate Management for Temporomandibular Disorders

1. Awareness of habits or jaw use patterns

The teeth are supposed to make contact during eating and swallowing, but at no other time. When the jaw is not working during eating, swallowing, yawning and talking, the teeth should be apart and the jaw should be at rest.

Notice any contact your teeth make.

Notice any positions your jaw continually returns to.

Notice any clenching, grinding, gritting, tapping of teeth or tensing of jaw muscles.

Notice when these tooth contacts or the jaw muscle tensing most often occurs such as during driving, study, reading, social situations, conversation, fatigue, overwork, stress, emotional upsets, work, sports.

Be aware of whether you are able to eat on both sides.

2. Positioning of the jaw to avoid tooth contacting habit

Place the tip of the tongue just behind the top front teeth and keep the teeth slightly apart. Maintain this position whenever the jaw is not being used.

3. Diet Modifications

Softer foods place less stress on the jaw muscles and joints than coarser foods.

Avoid eating coarse, hard foods that require you to bite into them with the front teeth, such as apples, or sandwiches. Cut these foods up into small pieces and eat them on the back teeth.

Do not chew gum or soft chewy foods that require excessive jaw movements.

4. Avoid wide jaw openings

Excessive movements of the jaw will place stress on the joint and the muscles.

5. DO NOT TEST THE JAW!

You may want to periodically move your jaw around to check whether you are making progress and to see if the soreness is resolving. To do this, people usually open and swing the jaw from side to side beyond the comfortable range of motion. When you move your jaw to the point where you produce pain and discomfort, you have added to the stress on your muscles and joints and this can cause continuation of the problems you are trying to eliminate.

6. Sleep Patterns

Avoid sleeping on your stomach which places the jaw in a position where considerable pressure is placed upon it. Propping pillows beside you may be helpful in avoiding unconsciously moving onto your stomach. This can be a difficult pattern to break.

Avoid sleeping positions where the jaw is rested upon a hand or arm which also applies considerable pressure to the jaw.

7. Support the jaw during yawning

Place your index finger and thumb on your chin to provide some extra stability to the lower jaw during yawning.

8. Incidental

Avoid leaning on your chin or jaw at any time. This even applies to posturing your head to press a telephone against your shoulder.

There can be an indefinite number of factors contributing to jaw dysfunction. It is easily possible that this guide has overlooked a factor that is important in your problem. Please advise us of anything that you think may be contributing. Your input is important.

9. Use of moist heat or ice

Moist heat: Place a warm damp towel along the side of your face. Place a hot water bottle over the top of the damp towel. Maintain this for 15–20 minutes.

Ice: Place a gel pack or bag of frozen peas or corn along the side of your face (do not place cold pack directly on your skin, always use a paper towel or thin cloth next to your skin). Maintain this for 15–20 minutes.

Generally, Ice is applied for an acute injury (a recent injury which often produces severe pain and swelling). The effect of ice application is to reduce pain and swelling (it has an analgesic, or numbing effect).

Heat is applied for pain reduction and muscle relaxation, and is used for non-acute conditions.

10. General Recommendations

Get adequate sleep.

Maintain good nutrition.

Try to avoid taking on additional stressful commitments that may adversely affect your present problem or that would infringe on your ability to manage your jaw problem.
