# Supplementary material for: Yoga for myofascial pain of masticatory muscles – a development and feasibility study
Source: BDJ Open. 2025 Dec 10;11:94. doi: 10.1038/s41405-025-00377-x (PMC12696018; doi:10.1038/s41405-025-00377-x)
Supplement: Supplementary file 3 — Appendix 2 [file 41405_2025_377_MOESM3_ESM.pdf]

Appendix 2: Schedule for Active Control and Yoga group, Yoga class content and walking program

Schedule for Active Control and Yoga group

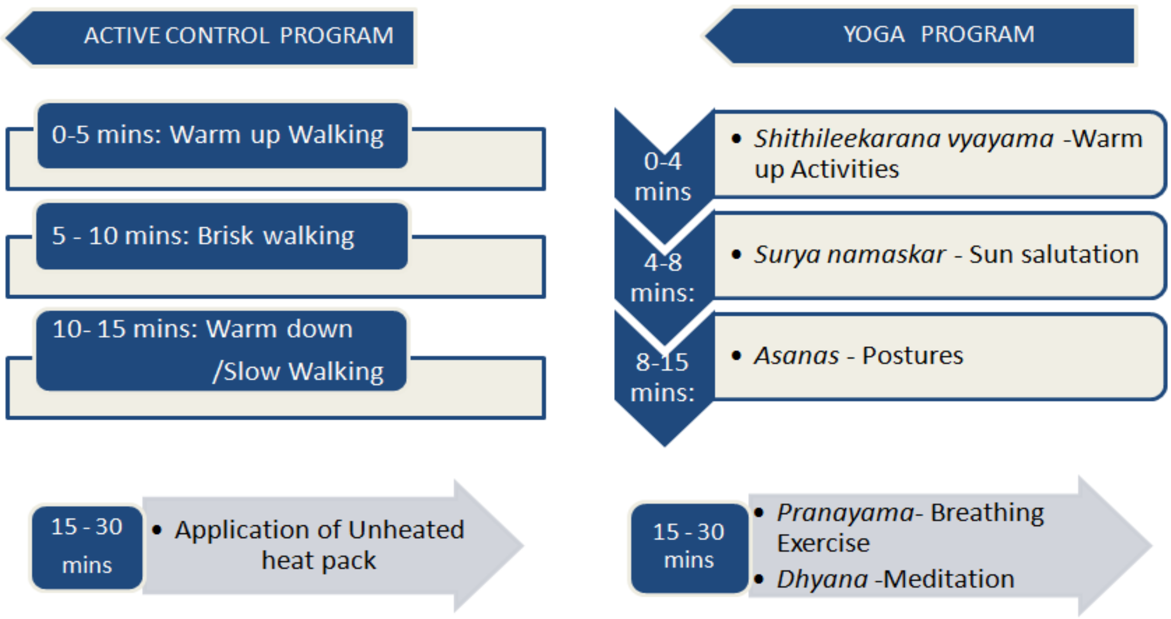

Yoga class content for week 1

WEEK 1 - YOGA PROGRAM

EVENT	TYPE OF MOVEMENT	No.of Breaths/No. of repetitions * duration	Time in mins.
**WARM UP ACTIVITIES**	<u>Shoulder movements</u> :<br>1. Rotation clockwise and anticlockwise<br>2. Horizontal Adduction and Abduction(flexion and extension)<br><u>Spinal twist</u><br><u>lateral twist</u><br><u>Upward dog with neck flexion and extension, Right and left lateral rotation</u>	10 breathing cycle (5 in clockwise and 5 in anticlockwise)<br>5 breathing cycles<br>3 breathing cycles<br>3 breathing cycles<br>4 breathing cycles (2 for neck flexion and extension; 2 for lateral rotation)	3- 4 mins.
**SUN SALUTATION**	short sequence involving 9 postures	4 shot sequence	4
	Position 1 (*Pranamasana* -Prayer pose)<br>Position 2 (*Hastha Uthanasana* -Raised arm pose)<br>Position 3 (*Padahastanasana* -Hand to Foot Pose)<br>Position 4 (*Aswa sanchalanasana* -Equestrian Pose)<br>Position 5 (*Parvatasana/Adhomuka svanasana* -Mountain pose/ Downward stretch dog)<br>Position 6 (*Aswa sanchalanasana* -Equestrian Pose)<br>Position 7 (*Padahastanasana* -Hand to Foot Pose)<br>Position 8 (*Hastha Uthanasana* -Raised arm pose)<br>Position 9 (*Pranamasana* -Prayer pose)		
**POSTURES**	*Utkatasana* - Chair posture<br>*Vakrasana* - Spinal twist<br>Variation of *Yoga mudr* a - Downward child posture with arm extension<br>*Marjariasana* - Cat posture	2* 30 sec<br>4*30 sec<br>3*40 sec<br>4*30 sec	1<br>2<br>2<br>2
Neck exercise	1.Clockwise and anticlockwise rotation<br>2. Right and Left lateral flexion		1<br>1
**RELAXATION**	Shavasana - corpse pose		1
**BREATHING EXERCISES**	Deep Breathing<br>*Anuloma Viloma* - Alternate nostril Breathing<br>*Mukhasana* - Facial breathing activity<br>*Anuloma Viloma* - Alternate nostril Breathing	5 cycles<br>3 cycles<br>3 cycles<br>2 rounds	1<br>2<br>4<br>2
**MEDITATION**	Breath Guided awareness		3

Yoga class content for week 2

WEEK 2 - YOGA PROGRAM

EVENTS	TYPE OF MOVEMENT	No.of Breaths/No. Of repetitions * duration	Time in mins.
	Shavasana - corpse pose		1
**RELAXATION**	Deep Breathing	5 cycles	1
**WARM UP ACTIVITIES**	Shoulder movements :		
	1. Rotation clockwise and anticlockwise	10 breathing cycle (5 in clockwise and 5 in anticlockwise)	
	2. Horizontal Adduction and Abduction(flexion and extension)	5 breathing cycles	
	Spinal twist	3 breathing cycles	
	lateral twist	3 breathing cycles	
	Upward dog with neck flexion and extension, Right and left lateral rotation	4 breathing cycles (2 for neck flexion and extension; 2 for lateral rotation)	3- 4 mins.
**SUN SALUTATION**	Short version and **Full Version (given in bold face type)**		
	Position 1 (*Pranamasana* -Prayer pose)		
	Position 2 (*Hastha Uthanasana* -Raised arm pose)		
	Position 3 (*Padahastanasana* -Hand to Foot Pose)		
	Position 4 (*Aswa sanchalanasana* -Equestrian Pose)		
	Position 5 (*Adhomuka svanasana* - Downward stretch dog)		
	**Position 6 (*Nirkunjasana* -Ashtanga namaskar)**	1 short sequence	
	**Position 7 (*Bhujangasana* -Serpent pose)**	1 full sequence	
	**Position 8 (Adhomuka svanasana-Downward stretch dog)**		
	Position 9 (*Aswa sanchalanasana* -Equestrian Pose)	1 short sequence	
	Position 10 (*Padahastanasana* -Hand to Foot Pose)		
	Position 11 (*Hastha Uthanasana* -Raised arm pose)		
	Position 12 (*Pranamasana* -Prayer pose)		4
**POSTURES**	*Utkatasana* - Chair posture	2* 30 sec	1
	Bharadvajasana - torso twist	4*30 sec	2
	Variation of Yoga mudra - Downward child posture with	3*40 sec	2
	Marjariasana - Cat posture	4*30 sec	2
**BREATHING EXERCISES**	*Bhastrika* - Rapid/Fast Breathing	3 cycles	2
	Mukhasana - Facial breathing	3 cycles	4
	Nadi Shodana -Alternate nostril with retention (1:1:1)	2 rounds	2
**MEDITATION**	Breath Guided awarness + modified Kechari mudra (Rolling of tongue towards soft palate)		3

Yoga class content for week 3 and 4

WEEK 3 & 4 - YOGA PROGRAM

EVENTS	TYPE OF MOVEMENT	No.of Breaths/No. Of repetitions * duration	Time in mins.
Centering Practices before start of Yoga	*Shavasana - corpse pose*		1
	Deep Breathing	5 cycles	1
	Anuloma Viloma - Alternate nostril Breathing	3 cycles	1
**WARM UP ACTIVITIES**	Shoulder movements :	10 breathing cycle	
	1. Rotation clockwise and anticlockwise	(5 clockwise, 5 anticlockwise)	
	2. Horizontal Adduction and Abduction(flexion and extension)	5 breathing cycles	
	Spinal twist	3 breathing cycles	
	lateral twist	3 breathing cycles	
	Upward dog with neck flexion and extension, Right and left lateral rotation	4 breathing cycles (2 for neck flexion and extension; 2 for lateral rotation)	3- 4 mins.
**SUN SALUTATION**	Short version and **Full Version (given in bold face type)**		
	Position 1 (*Pranamasana* -Prayer pose)		
	Position 2 (*Hastha Uthanasana* -Raised arm pose)		
	Position 3 (*Padahastanasana* -Hand to Foot Pose)		
	Position 4 (*Aswa sanchalanasana* -Equestrian Pose)		
	Position 5 (*Adhomuka svanasana* - Downward stretch dog)		
	**Position 6 (*Nirkunjasana* -Ashtanga namaskar)**	1 short sequence	
	**Position 7 (*Bhujangasana* -Serpent pose)**	3 full sequence	
	**Position 9 (*Adhomuka svanasana* -Downward stretch dog)**		
	Position 9 (*Aswa sanchalanasana* -Equestrian Pose)	1 short sequence	
	Position 10 (*Padahastanasana* -Hand to Foot Pose)		
	Position 11 (*Hastha Uthanasana* -Raised arm pose)		
	Position 12 (*Pranamasana* -Prayer pose)		4
**POSTURES**	*Tadasana* - Mountain posture	2* 30 sec	1
	Bharadvajasana - torso twist	4*30 sec	2
	Matsyasana - Fish posture	3*40 sec	2
	Urdhva Janu sirasana - Upward facing single leg forv	4*30 sec	2
**BREATHING EXERCISES**	*Nadi Shodana* -Alternate nostril with retention (1:2:1	3 cycles	2
	Mukhasana - Facial breathing	3 cycles	4
	Shanmukhi mudra - Seal of sense organ	2 rounds	2
	Bhramari - Humming Bee breath	3 cycles	2
**MEDITATION**	*'OM' kara* - OM Meditation		3

Further details and particulars of yoga activities are available in ANZCTR trial registration under supporting document as Appendix 1.

https://www.anzctr.org.au/Trial/Registration/TrialReview.aspx?id=383052&showOriginal=true&isReview=true

Walking program

Lap I: Walking at an average pace reaching 500 steps/ 0.38 km in 4.5 mins.

4.5 - 5 mins : 30 seconds rest

Lap II (5 - 10 mins): Walking at moderate intensity reaching 600 steps/0.45 km in 5 min

Lap III (10- 14 mins): Gradually reducing walking intensity covering 400 steps/0.3km in 4 mins.

14 - 15 mins: 1 minute rest
